# Genetic variation underlying renal uric acid excretion in Hispanic children: the Viva La Familia Study

**DOI:** 10.1186/s12881-016-0366-3

**Published:** 2017-01-17

**Authors:** Geetha Chittoor, Karin Haack, Nitesh R. Mehta, Sandra Laston, Shelley A. Cole, Anthony G. Comuzzie, Nancy F. Butte, V. Saroja Voruganti

**Affiliations:** 1 Department of Nutrition and UNC Nutrition Research Institute, University of North Carolina at Chapel Hill, 500 Laureate Way, Kannapolis, NC 28081 USA; 2 Department of Genetics, Texas Biomedical Research Institute, San Antonio, TX USA; 3 USDA/ARS Children’s Nutrition Research Center, Department of Pediatrics, Baylor College of Medicine, Houston, TX USA; 4 South Texas Diabetes and Obesity Institute, School of Medicine, University of Texas Rio Grande Valley, Brownsville, TX USA

**Keywords:** Uric acid clearance, Genetic variation, Fractional excretion of uric acid, Hispanic children

## Abstract

**Background:**

Reduced renal excretion of uric acid plays a significant role in the development of hyperuricemia and gout in adults. Hyperuricemia has been associated with chronic kidney disease and cardiovascular disease in children and adults. There are limited genome-wide association studies associating genetic polymorphisms with renal urate excretion measures. Therefore, we investigated the genetic factors that influence the excretion of uric acid and related indices in 768 Hispanic children of the Viva La Familia Study.

**Methods:**

We performed a genome-wide association analysis for 24-h urinary excretion measures such as urinary uric acid/urinary creatinine ratio, uric acid clearance, fractional excretion of uric acid, and glomerular load of uric acid in SOLAR, while accounting for non-independence among family members.

**Results:**

All renal urate excretion measures were significantly heritable (*p* <2 × 10^−6^) and ranged from 0.41 to 0.74. Empirical threshold for genome-wide significance was set at *p* <1 × 10^−7^. We observed a strong association (*p* < 8 × 10^−8^) of uric acid clearance with a single nucleotide polymorphism (SNP) in zinc finger protein 446 (*ZNF446*) (rs2033711 (A/G), MAF: 0.30). The minor allele (G) was associated with increased uric acid clearance. Also, we found suggestive associations of uric acid clearance with SNPs in *ZNF324*, *ZNF584*, and *ZNF132* (in a 72 kb region of 19q13; *p* <1 × 10^−6^, MAFs: 0.28–0.31).

**Conclusion:**

For the first time, we showed the importance of 19q13 region in the regulation of renal urate excretion in Hispanic children. Our findings indicate differences in inherent genetic architecture and shared environmental risk factors between our cohort and other pediatric and adult populations.

**Electronic supplementary material:**

The online version of this article (doi:10.1186/s12881-016-0366-3) contains supplementary material, which is available to authorized users.

## Background

Renal excretion of uric acid is commonly involved in the development of gout, hyperuricemia, and nephropathy [1, 2]. The kidney filters freely circulating uric acid, accounting for ~70% of total uric acid excretion from the body [3]. The prevalence of hyperuricemia (increased serum uric acid (SUA) concentrations) and gout are on the rise along with other metabolic disorders such as obesity, type 2 diabetes, and metabolic syndrome [3, 4]. Hyperuricemia and hyperuricosuria (increased urinary uric acid (UrUA) concentrations) can lead to uric acid nephrolithiasis [5–7]. Moreover, these two are common multifactorial disorders that have been shown to have a familial inheritance and further associated with progression to chronic kidney disease [4, 8, 9]. Hyperuricemia is shown to cluster within families, heritabilities ranged from 39 to 45% in our family studies [10, 11], and twin studies up to 80% for serum uric acid [12], 60% for renal clearance of urate and 87% for fractional excretion of urate [13].

Uric acid is the end-product of purine metabolism, which is secreted, filtered, and reabsorbed with the help of specific urate transporters such as uric acid transporter-1 protein (URAT1) and solute carrier family 2, member 9 (SLC2A9) [4, 14, 15]. Any defect in these urate transporters, affecting the secretion and reabsorption processes, might lead to increased concentration in blood or reduced excretion of uric acid in the body [3, 9]. We have shown that variation in SUA concentrations is affected by genetic factors and is also associated with obesity and its comorbidities in Hispanic children [11]. However, there are no extensive genetic studies on parameters of renal urate excretion in children. Therefore, assessing the genetic contribution to excretion of uric acid and its related indices might help develop methods to better understand the renal urate excretion in children. In the present study, our aim was to identify genetic loci contributing to renal urate excretion measures using the genome-wide association (GWA) approach in Hispanic children of Viva La Familia Study (VFS).

## Methods

### Viva La Familia Study (VFS)

VFS was designed to identify genetic variants influencing pediatric obesity and its comorbidities in Hispanic children with the majority from Mexican American families. VFS study design, recruitment, and methodology, demographic and phenotypic information have been described in detail elsewhere [16]. All participants gave written informed consent or assent. Institutional Review Boards at Baylor College of Medicine and Affiliated Hospitals, Texas Biomedical Research Institute and UNC Chapel Hill approved the protocol for Human Subject Research. Methods used to measure fasting blood and 24-h urinary biochemistries are described elsewhere [11]. Availability of the phenotypic data after calculation of urinary indices was limited to 768 children.

### Urinary and other indices calculation

Urine sample was collected over a 24-h period and were alkalized (pH around 8.0) using 0.5N NaOH during initial dilution. The urinary indices, using serum and urinary uric acid and creatinine, were calculated according to Perez-Ruiz, et al. [2] with following equations:

$$ \begin{array}{l}\mathrm{Body}\ \mathrm{S}\mathrm{urface}\ \mathrm{A}\mathrm{rea}\ \left(\mathrm{B}\mathrm{S}\mathrm{A},\ {\mathrm{m}}^2\right)\ \mathrm{calculated}\ \mathrm{b}\mathrm{y}\ \mathrm{Dubois}\ \mathrm{Equation} = 0.007184 \times \mathrm{Height}\ {\left(\mathrm{cm}\right)}^{0.725} \times \mathrm{Weight}\ {\left(\mathrm{kg}\right)}^{0.425}\hfill \\ {}\mathrm{Creatinine}\ \mathrm{clearance}\ \left(\mathrm{CrCl},\ \mathrm{ml}/ \min \right) = \mathrm{U}\mathrm{v} \times \left(\mathrm{UrCr}/\mathrm{SrCr}\right);\ \mathrm{U}\mathrm{v}:\ 24-\mathrm{hr}\ \mathrm{urine}\ \mathrm{v}\mathrm{o}\mathrm{lume}/\mathrm{time},\ \mathrm{U}\mathrm{rCr}:\ \mathrm{urinary}\ \mathrm{creatinine},\ \mathrm{S}\mathrm{rCr}:\ \mathrm{s}\mathrm{erum}\ \mathrm{creatinine}\hfill \\ {}\mathrm{U}\mathrm{ric}\ \mathrm{a}\mathrm{cid}\ \mathrm{clearance}\ \left(\mathrm{UACl},\ \mathrm{ml}/ \min \right) = \mathrm{U}\mathrm{v} \times \left(\mathrm{UrUA}/\mathrm{SrUA}\right);\ \mathrm{U}\mathrm{rUA}:\ \mathrm{urinary}\ \mathrm{uric}\ \mathrm{a}\mathrm{cid},\ \mathrm{S}\mathrm{rUA}:\ \mathrm{s}\mathrm{erum}\ \mathrm{uric}\ \mathrm{a}\mathrm{cid}\hfill \\ {}\mathrm{U}\mathrm{rinary}\ \mathrm{uric}\ \mathrm{a}\mathrm{cid}\ \mathrm{t}\mathrm{o}\ \mathrm{urinary}\ \mathrm{creatinine}\ \mathrm{ratio} = \mathrm{U}\mathrm{rUA}/\mathrm{UrCr}\ \mathrm{a}\mathrm{nd}\ \mathrm{expressed}\ \mathrm{a}\mathrm{s}\ \mathrm{a}\mathrm{b}\mathrm{s}\mathrm{o}\mathrm{lute}\ \mathrm{v}\mathrm{a}\mathrm{lue}\hfill \\ {}\mathrm{Fractional}\ \mathrm{excretion}\ \mathrm{o}\mathrm{f}\ \mathrm{uric}\ \mathrm{a}\mathrm{cid}\ \left(\mathrm{FEUA},\ \%\right) = \left(\mathrm{UrUA} \times \mathrm{S}\mathrm{rCr}\right)/\left(\mathrm{SrUA} \times \mathrm{U}\mathrm{rCr}\right) \times 100\hfill \\ {}\mathrm{Glomerular}\ \mathrm{load}\ \mathrm{o}\mathrm{f}\ \mathrm{uric}\ \mathrm{a}\mathrm{cid}\ \left(\mathrm{GLUA},\ \mathrm{mg}/ \min /{\mathrm{m}}^2\right) = \mathrm{CrCl} \times \mathrm{S}\mathrm{rUA}\hfill \\ {}\mathrm{Excretion}\ \mathrm{o}\mathrm{f}\ \mathrm{uric}\ \mathrm{a}\mathrm{cid}\ \mathrm{per}\ \mathrm{v}\mathrm{o}\mathrm{lume}\ \mathrm{o}\mathrm{f}\ \mathrm{glomerular}\ \mathrm{f}\mathrm{iltration}\ \left(\mathrm{EUAGF},\ \mathrm{mg}/\mathrm{dL}/{\mathrm{m}}^2\right) = \left(\mathrm{UrUA} \times \mathrm{S}\mathrm{rCr}\right)/\mathrm{UrCr}\hfill \end{array} $$

### SNP genotyping

The Illumina HumanOmni1-Quad v1.0 BeadChip marker assays were used to genotype 1.1 million SNPs in 815 children enrolled in VFS [17]. Genotype calls were obtained after scanning on the Illumina BeadStation 500GX and analyzed by using the GenomeStudio software. Genotyping error rate was 2 per 100,000 genotypes (based on duplicates). The average call rate for all SNPs per individual sample was 97%. SNP genotypes were checked for Mendelian consistency using the program, SimWalk2 [18]. The estimates of the allele frequencies and their standard errors were obtained using SOLAR [19].

### Heritability analysis

A variance components decomposition method was used to estimate heritability of uric acid and other renal urate excretion phenotypes. To estimate the genetic contribution to the variation in urinary indices, their heritability was estimated in SOLAR. Total phenotypic variance can be partitioned into its genetic and environmental components. The fraction of total phenotypic variance (*VP*) resulting from additive genetic effects (*VG*) is called heritability and is denoted as *h2* = *VG/VP* [20]. All traits were adjusted for age, sex, their interaction effects, and body surface area.

### Genome-wide association (GWA) study of urinary uric acid excretion measures

A total of 899,892 SNPs passed quality control and were included in the GWA analysis. GWAS was performed on 768 children from 260 Hispanic families including 1643 relative pairs. A measured genotype analysis (MGA) was performed on the inverse normal transformed residual traits (after regressing for covariate effects mentioned above) to minimize the non-normality distribution of the data using SOLAR. Each SNP genotype was converted in SOLAR to a covariate measure equal to 0, 1, or 2 copies of the minor alleles (or, the weighted covariate based on imputation for missing genotypes). These SNP covariates also were included in the variance components mixed models for MGA [21] vs. null models that incorporated the random effect of kinship and fixed effects such as age, sex, their interaction effects, and body surface area. For the initial GWA screen, we tested each SNP covariate independently as a 1-df likelihood ratio test. The linkage disequilibrium (LD) was computed in SOLAR by using information for all genotyped SNPs in all individuals. The effective number of SNPs accounting for LD was calculated by the method of Moskvina and Schmidt [22]. The average ratio of effective number of SNPs to the actual number obtained from analysis of non-overlapping bins of SNPs was used to calculate the genome-wide effective number of tests and thus the significance threshold for genome-wide association. Empirical thresholds for genome-wide significant and suggestive evidence of association were based on the distribution of *p*-values from 10,000 simulated null GWAS (i.e. simulations of a heritable trait with no modeled SNP covariate effects using the VFS pedigree and genotypes). The threshold for significance (*p* < 1 × 10^−7^) was defined as the cutoff for the lower 5% tails of the empirical distribution, and the threshold for suggestive evidence (*p* < 1 × 10^−6^) was the minimum *p*-value obtained not more than once per genome scan [11, 22].

## Results

### General characteristics of renal urate excretion measures

The study included data from 768 Hispanic children for the traits considered. General characteristics are given in Table 1 for both boys and girls and the total population, respectively. Mean age of boys and girls was approximately 12 years; body surface area (BSA) was higher (*p* <0.05) for boys (mean ± sd: 1.55 ± 0.40 m^2^ vs. 1.47 ± 0.33 m^2^) than girls, similar with BSA z-scores (0.33 ± 0.91 vs. 0.14 ± 0.76).

**Table 1:** General characteristics and renal urate excretion in Hispanic children

Variable	Boys (*N* = 397)	Girls (*N* = 371)	Total (*N* = 768)	Heritability estimates	
	Mean ± SD	Mean ± SD	Mean ± SD	h^2^ ± SE	*P* value
Age, years	11.88 ± 3.24	12.02 ± 3.48	11.95 ± 3.36	-	-
BSA, m^2^	1.55 ± 0.40*	1.47 ± 0.33*	1.51 ± 0.37	-	-
BSAZ	0.33 ± 0.91*	0.14 ± 0.76*	0.24 ± 0.85	-	-
UrCr, mg/dL	95.37 ± 39.53	83.86 ± 32.10	89.81 ± 36.57	0.41 ± 0.10	1.9 × 10^−06^
CrCl, ml/min	60.59 ± 35.11*	47.41 ± 26.20*	54.22 ± 31.80	0.52 ± 0.09	2.5 × 10^−10^
SUA, mg/dL	5.84 ± 1.76*	4.99 ± 1.43*	5.43 ± 1.66	0.63 ± 0.09	1.4 × 10^−12^
UrUA, mg/dL	15.63 ± 13.19*	13.43 ± 11.41*	14.57 ± 12.40	0.70 ± 0.05	1.1 × 10^−24^
UACl, ml/min	2.99 ± 2.31*	2.43 ± 1.96*	2.72 ± 2.17	0.65 ± 0.06	1.7 × 10^−20^
UrUA/UrCr	0.17 ± 0.11*	0.16 ± 0.11*	0.16 ± 0.11	0.73 ± 0.05	1.3 × 10^−25^
FEUA, %	6.02 ± 5.14	6.34 ± 6.02	6.17 ± 5.58	0.74 ± 0.05	6.7 × 10^−27^
GLUA, mg/min/m^2^	3.63 ± 2.64*	2.33 ± 1.46*	3.00 ± 2.25	0.57 ± 0.09	2.2 × 10^−11^
EUAGF, mg/dL/m^2^	0.33 ± 0.30	0.30 ± 0.31	0.32 ± 0.31	0.73 ± 0.05	6.1 × 10^−25^

*p-value is significantly different at <0.05 between boys and girls; *BSA* body surface area, *BSAZ* body surface area z-scores, *UrCr* urinary creatinine, *SUA* serum uric acid, *UrUA* urinary uric acid, *UACl* uric acid clearance, *UrUA/UrCr* urinary uric acid to urinary creatinine ratio, *FEUA* fractional excretion of uric acid, *GLUA* glomerular load of uric acid, *EUAGF* excretion of uric acid per volume of glomerular filtration. All traits for heritability analyses adjusted for age, sex, age*sex, age^2, age^2*sex, body surface area z-scores; *p*-value <0.05 considered statistically significant

Renal 24-h urate excretion measures and their descriptions are given in Table 1. Creatinine clearance (CrCl), serum uric acid (SUA), urinary uric acid (UrUA), uric acid clearance (UACl), urinary uric acid to urinary creatinine ratio (UrUA/UrCr), and glomerular load of uric acid (GLUA) were higher in boys than girls (*p* <0.05). However, urinary creatinine (UrCr), and excretion of uric acid per volume of glomerular filtration (EUAGF) tended to be higher in boys compared to girls; and, fractional excretion of uric acid (FEUA) tended to be higher in girls compared to boys, but did not reach statistical significance.

### Heritability estimates

Table 1 also lists the heritability estimates and corresponding *p*-values for the phenotypes considered in this study. All phenotypes were adjusted for covariate effects (age, sex, age*sex, age2, age2*sex, body surface area z-scores). Significant heritability (h^2^) was detected for all the traits (*p* <2 × 10^−6^) and ranged from 0.41 to 0.74.

### Genome-wide association analysis

**Fig. 1:**
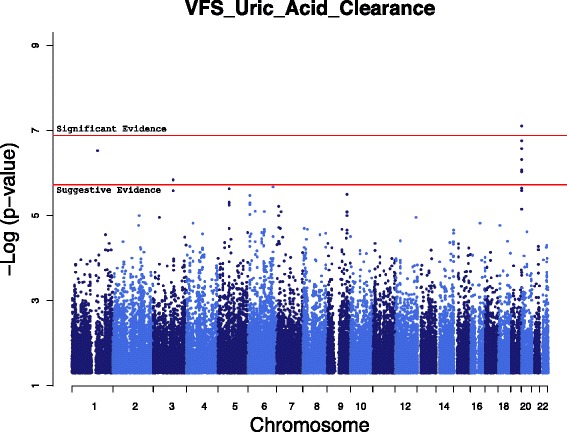
Genome-wide scan showing significant evidence of association for uric acid clearance with variants on chromosome 19

**Fig. 2:**
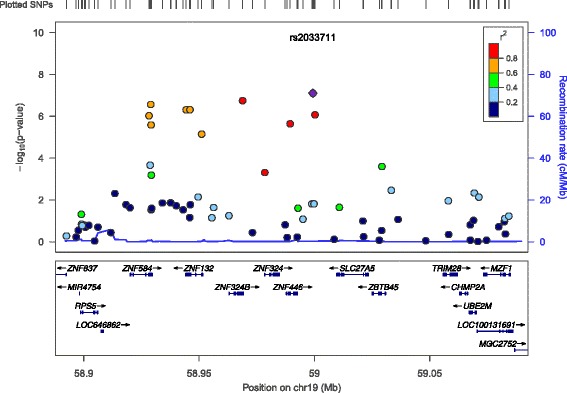
Locus Zoom plot showing the most significant SNPs on chromosome 19q13

Measured Genotype Analysis (MGA) was conducted using a variance components approach in 768 VFS Hispanic children that accounted for family kinships (Table 2). Genome-wide significant evidence of association was found for single nucleotide polymorphism (SNP) rs2033711, an intronic variant, in zinc finger protein 446 (*ZNF446*) on chromosome 19 with UACl (*p* <8 × 10^−8^, minor allele frequency (MAF) = 0.30) (Fig. 1). The effect size or the proportion of the residual phenotypic variance accounted for by the minor allele of the SNP was 4.5%. Genotype-specific means of this SNP showed that minor allele (G) was associated with increased UACl. In addition to *ZNF446*, a 72 kb region of chromosome 19q13 containing several zinc finger protein (*ZNF*) genes (*ZNF324*, *ZNF584* and *ZNF132*) showed suggestive association with UACl (Fig. 2 & Additional file 1: Table S1), but after accounting for linkage disequilibrium (LD; r^2^ < 0.80) only *ZNF446* and *ZNF584* and an insulin receptor-related receptor (*INSRR*) gene on chromosome 1q21-23 showed evidence of suggestive association with UACl (Table 2).

**Table 2:** Results of genome-wide association analyses of renal urate excretion measures in Viva Hispanic children

SNP	Phenotype	Chr	Pos (GRCh38)	p (SNP)	β	SE	Effect size (%)	MAF	Gene symbol
rs2033711	UACl	19	58487983	7.9 × 10^−08^	0.32	0.06	4.5	G (0.30)	*ZNF446*
rs10908521	UACl	1	56843858	3. 0 × 10^−07^	−0.35	0.07	4.3	G (0.21)	*INSRR*
rs10423138	UACl	19	58416935	9. 4 × 10^−07^	0.29	0.06	3.7	G (0.31)	*ZNF584*
rs9874872	UrUA/UrCr	3	31467743	1.8 × 10^−07^	−0.38	0.07	4.7	A (0.18)	
rs1353327	UrUA/UrCr	3	31461468	6. 8 × 10^−07^	−0.49	0.10	4.1	A (0.10)	
rs17079585	CrCl	13	23921903	2. 5 × 10^−07^	−1.53	0.30	4.0	A (0.007)	*ANKRD20A19P*
rs4889855	FEUA	17	80539480	2. 7 × 10^−07^	−0.29	0.06	4.9	G (0.43)	
rs17079585	GLUA	13	23921903	5. 7 × 10^−07^	−1.30	0.26	4.0	A (0.007)	*ANKRD20A19P*

*SNP* single nucleotide polymorphism, *Chr* Chromosome, *Pos* position in base pairs, *p(SNP) p*-value <1 × 10^−7^ shows evidence of significant association, *p* <1 × 10^−6^ shows evidence of suggestive association, *β* beta coefficient of the SNP, *SE* standard error of the beta coefficient; Effect Size: proportion of the residual phenotypic variance that is explained by the minor allele of the SNP; *MAF* minor allele frequency, *UACl* uric acid clearance, *UrUA/UrCr* urinary uric acid to urinary creatinine ratio, *CrCl* creatinine clearance, *FEUA* fractional excretion of uric acid, *GLUA* glomerular load of uric acid, *ZNF446*, zinc finger protein 446, *INSRR* Insulin receptor-related receptor, *ZNF584* zinc finger protein 584, *ANKRD20A19P* ankyrin repeat domain 20 family, member A19, pseudogene

A total of 7 SNPs were found to be suggestively (*p* <1 × 10^−6^) associated with one or the other renal urate excretion measures with MAFs ranging from 0.01 to 0.43, and the effect size ranged from 3.1 to 4.9%, respectively. After 19q13, another notable region where either GLUA or CrCl exhibited suggestive association was with a 13q12 SNP in ankyrin repeat domain 20 family, member A19, pseudogene (*ANKRD20A19P*). The genotype-specific mean values and the direction of associations (for minor alleles) of the SNPs in the genes associated with renal urate excretion measures from GWAS are shown in Table 3. For all UACl associated SNPs, the minor allele was associated with elevated UACl, except for rs10908521 on chromosome 1 (Additional file 2: Figure S1).

**Table 3:** Genotypic class specific mean values for top SNPs from genome-wide association analyses of renal urate excretion measures

SNP	Trait	Chr	Major/Major	Major/Minor	Minor/Minor	Direction of Change^a^
rs2033711	UACl	19	2.17 ± 1.79	2.79 ± 2.19	3.48 ± 2.69	↑
rs10908521	UACl	1	2.86 ± 2.28	2.06 ± 1.66	1.86 ± 1.34	↓
rs10423138	UACl	19	2.18 ± 1.78	2.77 ± 2.17	3.38 ± 2.68	↑
rs9874872	UrUA/UrCr	3	0.18 ± 0.12	0.13 ± 0.10	0.11 ± 0.08	↓
rs1353327	UrUA/UrCr	3	0.17 ± 0.11	0.11 ± 0.09	0.14 ± 0.13	↓
rs17079585	CrCl	13	54.26 ± 32.25	22.47 ± 14.47	-	↓
rs4889855	FEUA	17	7.22 ± 6.01	5.88 ± 5.74	4.34 ± 3.76	↓
rs17079585	GLUA	13	3.02 ± 2.28	1.28 ± 0.77	-	↓

^a^Direction of associations for minor alleles of the SNPs in the genes associated with renal handling measures from GWAS; *Chr* chromosome, *UACl* uric acid clearance, *UrUA/UrCr* urinary uric acid to urinary creatinine ratio, *CrCl* creatinine clearance, *FEUA* fractional excretion of uric acid, *GLUA* glomerular load of uric acid

## Discussion

This GWAS identified a set of genes encoding zinc finger proteins on chromosome 19q13 that were associated with uric acid clearance in Hispanic children. To the best of our knowledge, this is the first family-based GWAS of renal urate excretion measures with prominent effect sizes reported in children.

The kidney is responsible for ~70% of the excretion of total body uric acid, and also reabsorbs about 90% of the filtered urate, regulating circulating uric acid levels. Epidemiological studies have shown that optimum uric acid levels may have antioxidant properties, however, elevated SUA and UrUA levels are associated with hypertension, inflammation, and also with kidney stones [3, 7, 23, 24]. A urate-transporting molecular complex (urate transportome) has been proposed as a model for urate transport in renal proximal tubules since several membrane proteins seem to be involved in urate transport [25]. According to this model, the mechanism of uric acid transport cannot be understood by evaluating one or two transporters. It has to be investigated as a functional unit comprising of all uric acid transporters and other molecules. This is because the renal handling of urate transport involves several genes (e.g., solute carrier family 2, member 9 (*SLC2A9*) and ATP-binding cassette ABC, subfamily G, member 2 (*ABCG2*), solute carrier family 16, member 9 (*SLC16A9*), solute carrier family 17, members 1, 3 and 4 (*SLC17A1*, *SLC17A3* and *SLC17A4*), and, solute carrier family 22, members 11 and 12 (*SLC22A11* and *SLC22A12*), most of which have been implicated in the regulation of urate levels [26–29].

Defects in the tubular secretion of uric acid are mainly involved with urate clearance, hyperuricemia and gout [2]. Tubular secretion and reabsorption of urate levels change dynamically with age [9]. Genetic disorders have been indicated in the prevalence of hyperuricemia with increased urinary uric acid levels, which eventually lead to the formation of kidney stones and are often associated with chronic kidney disease [7, 30, 31]. Studies have demonstrated in adults that renal excretion of uric acid is under considerable genetic influence [32]. Our results indicated that all renal urate excretion measures are heritable and are within similar range of heritabilities reported in twin adults, [13] and Chinese twin children and adolescents [12].

Our GWAS findings on chromosome 19 revealed genes that have not been linked to renal urate handling before. We observed a strong genome-wide significant association of UACl with rs2033711 in *ZNF446*. Additionally, we found suggestive association of UACl with *ZNF584* genetic variant (rs10423138), not in LD with rs2033711 (*ZNF446*) suggesting that these genetic variants are independently influencing variation in UACl. The ZNF family is one of the major human gene families and comprises of several of the currently recognized transcription factors. ZNFs through the zinc finger motifs are shown to interact with nucleic acids and are involved in various molecular mechanisms, cell differentiation and development [33, 34]. Studies indicate that *ZNF446* is a novel member of Kruppel-related family, and believed to be one of the conserved proteins during human evolution. Also, *ZNF446* gene is highly expressed in adult tissue cells (muscle), and may function as a transcriptional repressor in cellular growth and development [34, 35]. It is known to inhibit transcriptional activities of serum response element (SRE) activator protein 1(AP-1) [34]. Interestingly, AP-1 is a *cis* regulatory element regulating *ABCG2* expression, one of the main uric acid transporter [36].

Moreover, strong association of UACl with *ZNF446* itself is interesting because studies have shown association of *ZNF365*, on chromosome 10, with urolithiasis in children, [7] and with uric acid nephrolithiasis in adults [5, 37]. However, our results indicate a different pattern of zinc finger protein involvement in uric acid metabolism compared with adult studies [5, 7, 37]. The functional relevance of these genes with uric acid involvement is largely unknown, except that the emergence of *ZNF365* variants is correlated with disappearance of uricase in primate evolution and hence causing a predisposition to hyperuricemia in humans [5, 37].

We found suggestive association of rs4889855 on chromosome 17 (Gene not identified) with FEUA. However, in adults, FEUA was shown to be associated with genetic variants of glucokinase regulator (*GCKR* on chromosome 2), *SLC2A9* and *ABCG2* on chromosome 4, and insulin like growth factor 1 receptor (*IGF1R* on chromosome 15) [38]. We also found suggestive evidence of association between GLUA and CrCl and *ANKRD20A19P* gene variant (on chromosome 13q12), both these associations prove to be interesting as beta-spectrin and ankyrin are key components of the cytoskeleton membrane that regulates clustering of sodium channel [39]. Additionally, we also found suggestive association of UACl with SNPs in *INSRR* on chromosome 1. Some studies have suggested functional association between mRNA expression of insulin receptor-related and insulin-like growth factor receptors and tumor cells [40]. One GWAS study indicated a new locus linked to chromosome 2p22.1-p21 for familial juvenile hyperuricaemic nephropathy, [41] but none of our associations with urinary uric acid handling have been reported before. The relatively small sample size in this study is a limitation. However, family-based studies have increased power to detect associations due to the fact that 768 children generate 1643 relative pairs and the degree of resemblance between relative pairs is considered for the genetic analysis.

Our study in VFS children is the first to report such genetic findings, and existing literature is limited in children involving GWAS on renal urate excretion measures. However, previous studies have reported association of 19q13 region with kidney phenotypes and diseases [42, 43]. This region has been linked to familial nephrotic syndrome [44], focal segmental glomerularosclerosis [45, 46] and cystinuria [47]. Our results are also different from studies that have reported association of uric acid transporters with renal uric acid excretion, for example, *ABCG2* for renal urate excretion [27], and *GCKR*, *SLC2A9*, and *IGF1R* for FEUA [38], partly attributable to population substructure and sample size considered as majority of the GWAS are conducted in European or Asian descent populations. Nevertheless, our 19q13 region contains several ZNF proteins (including *ZNF446*) that may be involved in transcription of specific uric acid transporters. Thus, our GWAS associations could reflect differences in inherent genetic architecture and shared environmental risk factors between our VFS pediatric cohort and other study populations.

## Conclusions

Our GWAS identified novel loci, particularly in 19q13, influencing the regulation of renal excretion of uric acid in Hispanic children. Our findings from children are not identical with those from adults [5, 37, 38] suggesting metabolic alterations in uric acid metabolism tracking from childhood to adults. The majority of the GWAS studies are conducted in adults highlighting the need for pediatric studies investigating the genetic underpinnings of the variation in uric acid earlier in the life course. It is essential to acquire knowledge on renal urate handling in children as it may reveal clinical and biological insights regarding the pathophysiology of uric acid excretion by the kidneys, given the inherited nature of these disorders.

## Additional files

Additional file 1:
**Table S1.** Results of genome-wide association analysis for uric acid clearance with variants on chromosome 19 in Viva Hispanic children. (DOCX 17 kb)
Additional file 2:
**Figure S1.** Graphical presentation of genotypic class specific mean values for top SNPs from GWAS of uric acid clearance. (GIF 40 kb)

## Abbreviations

*ABCG2*: ATP-binding cassette ABC, subfamily G, member 2
CrCl: Creatinine clearance
EUAGF: Excretion of uric acid per volume of glomerular filtration
FEUA: Fractional excretion of uric acid
*GCKR*: Glucokinase regulator
GLUA: Glomerular load of uric acid
GWAS: Genome-wide association study
*IGF1R*: Insulin like growth factor 1 receptor
*SLC2A9*: Solute carrier family 2, member 9
SrCr: Serum creatinine
SUA: Serum uric acid
UACl: Uric acid clearance
*URAT-1*: Uric acid transporter-1 protein
UrCr: Urinary creatinine
UrUA: Urinary uric acid
UrUA/UrCr: Urinary uric acid to urinary creatinine ratio
VFS: Viva La Familia Study
ZNF: Zinc Finger Proteins
