# Supplementary material for: Genetic variation underlying renal uric acid excretion in Hispanic children: the Viva La Familia Study
Source: BMC Med Genet. 2017 Jan 17;18:6. doi: 10.1186/s12881-016-0366-3 (PMC5240212; doi:10.1186/s12881-016-0366-3)
Supplement: Additional file 1: — Table S1. Results of genome-wide association analysis for uric acid clearance with variants on chromosome 19 in Viva Hispanic children. (DOCX 17 kb) [file 12881_2016_366_MOESM1_ESM.docx]

**Supplementary Table 1. Results of genome-wide association analysis for uric acid clearance with variants on chromosome 19 in Viva Hispanic children**

| **SNP^*^** | **Phenotype** | **Chr** | **Pos (GRCh38)** | **p(SNP)** | **β** | **SE** | **Effect Size (%)** | **MAF** | **Gene Symbol** |
| --- | --- | --- | --- | --- | --- | --- | --- | --- | --- |
| rs2033711 | UACl | 19 | 58487983 | 7.9 x 10^-08^ | 0.32 | 0.06 | 4.5 | G (0.30) | *ZNF446* |
| rs1077420 | UACl | 19 | 58457508 | 1.8 x 10^-07^ | 0.31 | 0.06 | 4.3 | C (0.29) | *ZNF324* |
| rs3764535 | UACl | 19 | 58417685 | 2.7 x 10^-07^ | 0.32 | 0.06 | 4.2 | A (0.28) | *ZNF584* |
| rs1051500 | UACl | 19 | 58433035 | 4.8 x 10^-07^ | 0.30 | 0.06 | 3.9 | G (0.31) | *ZNF132* |
| rs1465789 | UACl | 19 | 58434689 | 4.8 x 10^-07^ | 0.30 | 0.06 | 3.9 | A (0.31) | *ZNF132* |
| rs4801273 | UACl | 19 | 58488906 | 8.5 x 10^-07^ | 0.30 | 0.06 | 3.9 | G (0.29) | *ZNF446* |
| rs10423138 | UACl | 19 | 58416935 | 9. 4 x 10^-07^ | 0.29 | 0.06 | 3.7 | G (0.31) | *ZNF584* |

SNP: single nucleotide polymorphism; Chr: Chromosome; Pos: position in base pairs; p(SNP): p-value <1 x 10^-7^ shows evidence of significant association, p <1 x 10^-6^ shows evidence of suggestive association; β: beta coefficient of the SNP; SE: standard error of the beta coefficient; Effect Size: proportion of the residual phenotypic variance that is explained by the minor allele of the SNP; MAF: minor allele frequency; UACl: uric acid clearance; UrUA/UrCr: urinary uric acid to urinary creatinine ratio; CrCl: creatinine clearance; FEUA: fractional excretion of uric aicd; GLUA: glomerular load of uric acid; *ZNF446*: zinc finger protein 446; *ZNF324*: zinc finger protein 324; *ZNF584*: zinc finger protein 584; *ZNF132*: zinc finger protein 132

^*^rs2033711 and rs4801273 were in LD (r^2^ >0.80); and rs10423138 was in LD with rs1051500, rs1077420, rs1465789, and rs3764535
