# Supplementary figures and images for: Genetic variation underlying renal uric acid excretion in Hispanic children: the Viva La Familia Study
Source: BMC Med Genet. 2017 Jan 17;18:6. doi: 10.1186/s12881-016-0366-3 (PMC5240212; doi:10.1186/s12881-016-0366-3)

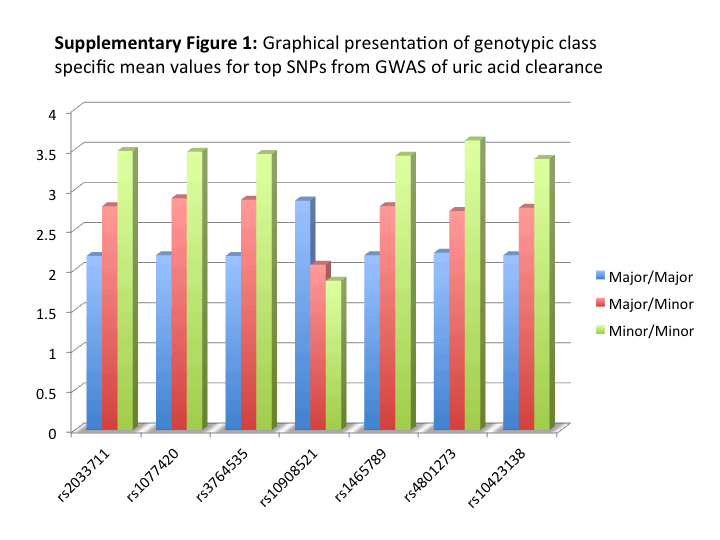

Supplement: Additional file 2: — Figure S1. Graphical presentation of genotypic class specific mean values for top SNPs from GWAS of uric acid clearance. (GIF 40 kb) [file 12881_2016_366_MOESM2_ESM.gif]
